# Konstantin Tsinman (1968-2020)

**DOI:** 10.5599/admet.1556

**Published:** 2022-12-06

**Authors:** Alex Avdeef

**Affiliations:** In-ADME Research, 1732 First Avenue #102, New York, NY 10128 USA; alex@in-adme.com

**Figure:**
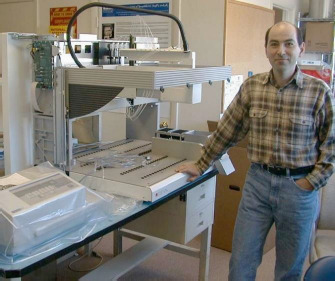


Dr. Konstantin Tsinman, Chief Scientific Officer at Pion Inc., succumbed after a three-week struggle, to the ravages of Covid-19 infection on Friday, 24 April 2020. Those of us who knew Konstantin were stunned to hear the horrible news. It is tragic to lose such a gifted scientist in the prime of his career, with so many valuable contributions to his credit and with so many yet to come. And to his wife Oksana and their daughter Tonia, what can anyone say to soften the deep pain and sorrow of loss?

I admired Konstantin very much. He had a calm demeanor, was very smart, unpretentious, very easily approachable. I still see his smiling face in my mind.

Konstantin joined Pion in 1998 (earliest photo of him above), just as the seedling company was starting to expand, poised to develop innovative scientific equipment for pharmaceutical research. The start-up secured its first external funds from a prominent pharmaceutical company in New Jersey, in exchange for the promise of delivery of the first “PAMPA” instruments (Parallel Artificial Membrane Permeability Assay). We had been working behind the scenes with Dr. Manfred Kansy of Roche (Basel), the inventor of PAMPA, for the preceding two years, before his famous seminal paper was published in 1998. So, that year we took the investment we secured and spent the whole lot on (i) a lease to a modest space on the second floor of a two-story industrial building, above Mike’s Gym, on the outskirts of Cambridge, Massachusetts, (ii) a Tecan robot workstation (pictured above – which several of us carried up the rickety wooden staircase in the elevatorless building), (iii) hiring a laboratory scientist (who later went on to medical school to become a physician), and (iv) hiring a scientific programmer – Konstantin. He was a PhD physicist interested in robotics programming, with a ‘can do just about anything’ attitude. He received his advanced degrees from the Institute of Metal Physics and the Dnepropetrovsk State University in Ukraine. He and his family had just then emigrated to America. His wife, Oksana, also a scientist, eventually joined the Pion team, and took over the running of the laboratory – a truly remarkable husband-wife duo at work.

The first few years were extremely exciting, albeit somewhat stressful. At the same time, many good ideas kept coming our way, which eventually led to new and innovative instrument products. We even were able to convince a group of scientists at the FDA to join us in a collaborative study to validate a newly-invented potentiometric method for measuring intrinsic solubility of drugs. The resultant publication in 2000 spurred a lot of interest in Pion’s products for pharmaceutical research, laying the groundwork for its future.

**Figure:**
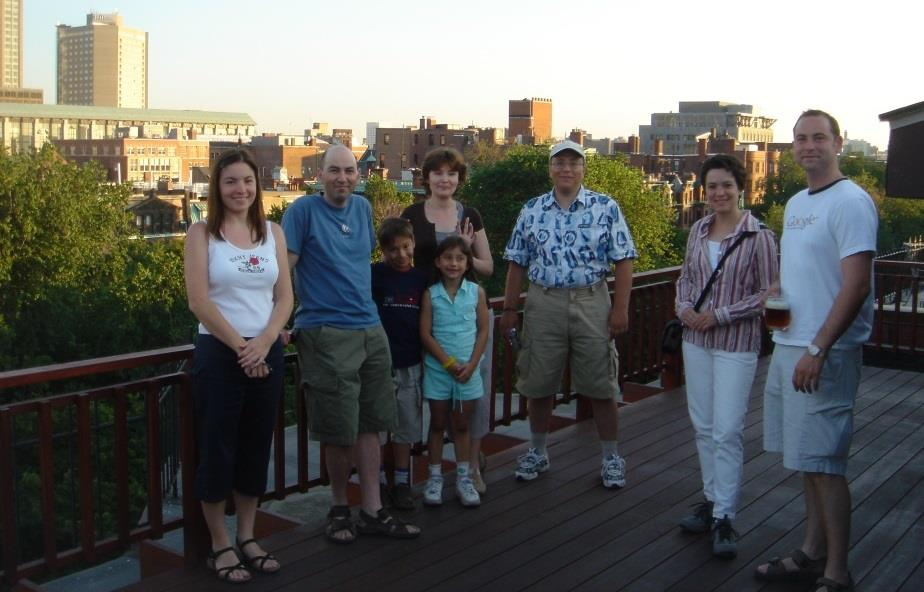
Relaxing with friends in the Back Bay of Boston on the fourth of July 2005. Konstantin is the second person from the left. Oksana is to his left. His life-long friend, Dima, is in the blue and white shirt near the center. Dima worked at Pion for many years.

Konstantin was the principal developer of the high throughput permeability analyzer and subsequently the high-throughput method for measuring solubility-pH profiles. He continued investigating physicochemical factors influencing intestinal absorption and penetration across the blood-brain-barrier of pharma research molecules, expanding the scope of applications for the UV fiber-optic technique. A long-standing challenge in drug dissolution measurement was the assessment of multicomponent active pharmaceutical ingredients in exploratory formulations. He developed the so-called “ZIM” technique elegantly to address the problem. Its later applications to studying nanoparticle suspensions were groundbreaking. It was such a clever idea that allowed the implementation of UV-Vis spectroscopy for real time concentration monitoring of complex phenomena.

Konstantin had been involved in multiple collaborative research projects with scientists from pharmaceutical companies and academic institutions, expanding the scope of applications for Pion's in situ UV fiber-optic dissolution technology, eventually coupling it with permeability measurement. He co-authored many articles in primary scientific journals and held several patents.

Konstantin was more than a laboratory researcher. He jumped right in to participate and lead in user training programs. He frequently traveled with the sales force to demonstrate products. He was an excellent speaker at scientific symposia all around the world. One could find him at the Pion booths at tradeshows, big and small. He always had a welcoming smile.

**Figure:**
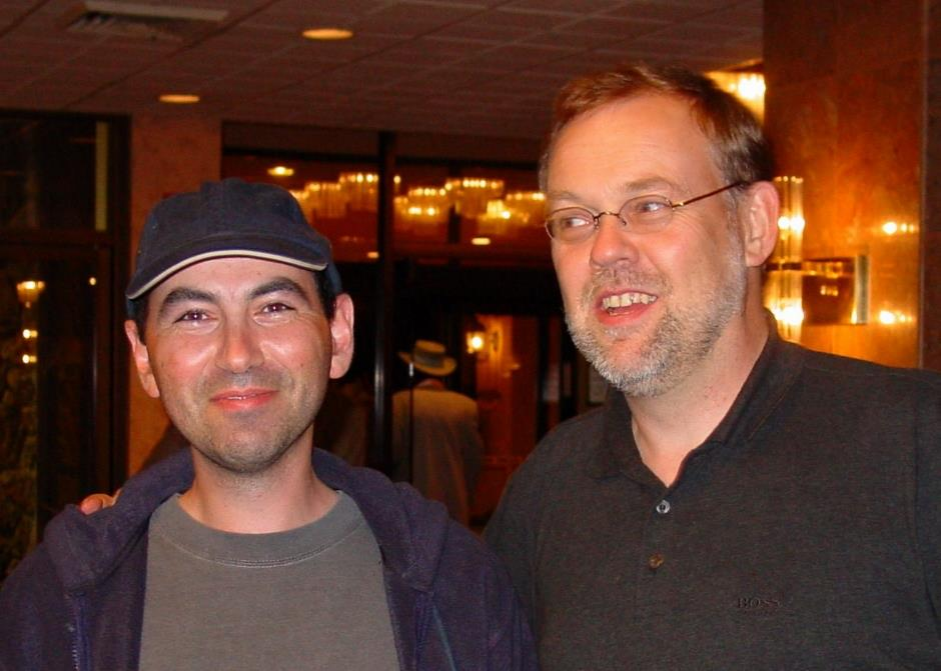
Konstantin with Manfred Kansy at the First International PAMPA Conference in San Francisco, California in 2002.

**Figure:**
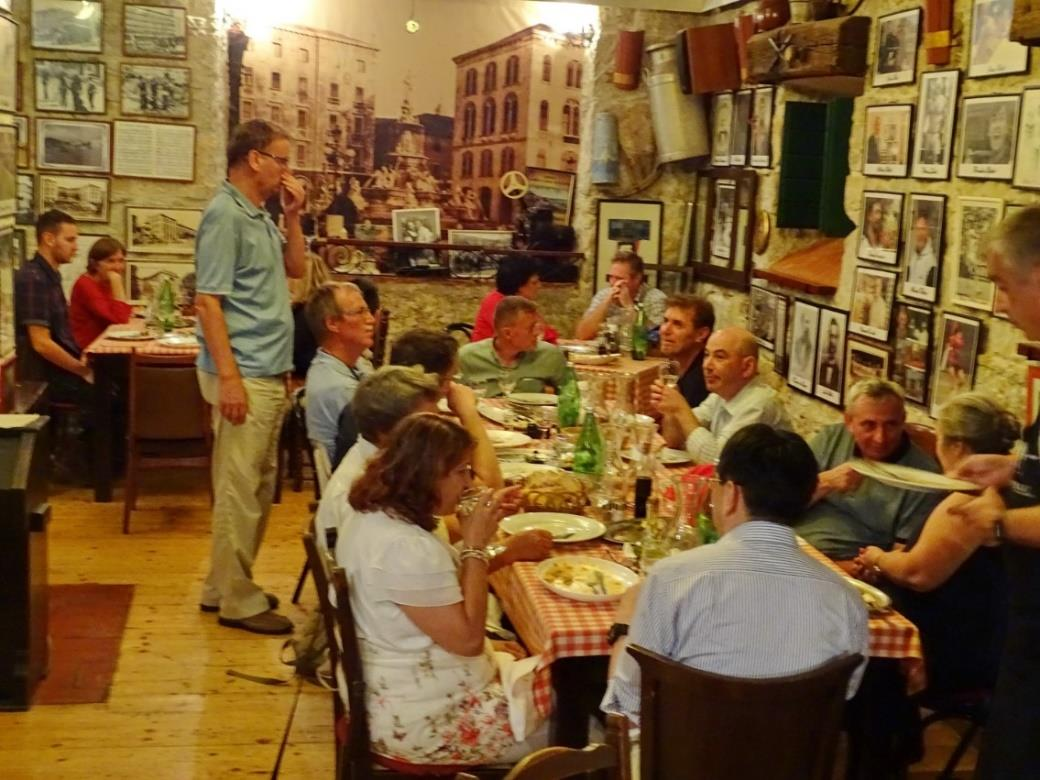
The last photo I have of Konstantin – the scientist amid scientists – in a quaint restaurant in the ancient Adriatic port of Split, Croatia, at the IAPC-8 meeting, September 2019. There were many other tables around the corner and there were musicians serenading and electrifying the spirits. The man standing is Professor Zoran Mandić (University of Zagreb), the seemingly tireless organizer of the IAPC series of conferences on topics related to drug discovery and development.

As news of Konstantin’s passing spread, I received many emails from those who knew him. A sampling of thoughts:

“I was shocked to hear the bad news, about the tragic death of Konstantin. It is incredible, such a young person as a victim of this virus pandemic. … I know … how bright a scientist he was. I met him first last year in Split and found a very nice guy and excellent speaker. I am very sorry… I am sitting and thinking on the life. It is so fragile.”

“Very shocked … such a good person.”

“I am really shocked and feel so sorry for the family. I remember the 4th of July on the roof in Boston so well. A happier time. Every day is precious…”

“I'm shocked … I met him in Boston last October. He has been very kind to me since I first met him.”

“… terribly sad news regarding Konstantin. I can’t believe it, he will be very sadly missed… I travelled with him on many occasions visiting customers and at trade shows. He was great company and taught me a lot about the science behind the pION products. Please pass on my deepest sympathy.”

“It is very sad news. Although I didn't know him well, I met him on several occasions and always had pleasant and sensible discussions.”

“My dear dear friend Konstantin, I´m out of words…Konstantin was one of the reasons for going to the AAPS, having chats and laughs, friendly but spot-on scientific discussions. The one who took care of me when arriving to pION and Boston in 1998 as a visiting PhD student …just starting my research career. I am devastated. So so sorry. What a big loss to this world - he will be so missed, for his sharp mind and friendliness.”

Konstantin will be deeply missed by his many friends. His soft-spoken, upbeat and cheerful manner endeared him to all with whom he had contact. He was an explorer, he was a teacher, and he was an inspiration to all.

*Искра жизни драгоценна и может быть мимолетной. Покойся с миром, друг мой.*

New York, November 2022.

